# Erratum: Recursive fury: conspiracist ideation in the blogosphere in response to research on conspiracist ideation

**DOI:** 10.3389/fpsyg.2013.00218

**Published:** 2013-04-12

**Authors:** Frontiers in Psychology Editorial Office

**Affiliations:** FrontiersLausanne, Switzerland

A correction implemented in the PDF file of the article was missed out in the HTML. Hence the PDF and full text HTML versions do not correspond. Under the heading *Survey responses “scammed” (1)* the following sentence: The notion of “scamming” took center-stage in the blogosphere's response to LOG12, although not all comments went so far as to suggest “… there are no ‘Human Subjects^4^.’ ” should read “The notion of ‘scamming’ took center-stage in the blogosphere's response to LOG12.” and footnote 4 should have been removed.

